# Supplementary material for: A functional role of meningeal lymphatics in sex difference of stress susceptibility in mice
Source: Nat Commun. 2022 Aug 16;13:4825. doi: 10.1038/s41467-022-32556-x (PMC9381547; doi:10.1038/s41467-022-32556-x)
Supplement: Supplementary file 1 — Supplementary Information [file 41467_2022_32556_MOESM1_ESM.pdf]

Supplementary Information

Title: A functional role of meningeal lymphatics in sex difference of stress susceptibility in mice

Authors: Weiping Dai[1,2,3†], Mengqian Yang[2,3†], Pei Xia[2,3†], Chuan Xiao[1,3,4], Shuying Huang[2,3], Zhan Zhang[1,3,4], Xin Cheng[2,3,], Wenchang Li[5], Jian Jin[6], Jingyun Zhang[2,3], Binghuo Wu[7], Yingying Zhang[2,3], Pei-hui Wu[5], Yangyang Lin[3,8], Wen Wu[6], Hu Zhao[2,3], Yan Zhang[9*], Wei-Jye Lin[1,3,4*], Xiaojing Ye[2,3*]

Affiliations:

1. Brain Research Center, Sun Yat-sen Memorial Hospital and Zhongshan School of Medicine, Sun Yat-sen University, Guangzhou, China

2. Faculty of Forensic Medicine, Guangdong Province Translational Forensic Medicine Engineering Technology Research Center, Zhongshan School of Medicine, Sun Yat-sen University, Guangzhou, China

3. Guangdong Province Key Laboratory of Brain Function and Disease, Zhongshan School of Medicine, Sun Yat-sen University, Guangzhou, China

4. Guangdong Provincial Key Laboratory of Malignant Tumor Epigenetics and Gene Regulation, Guangdong-Hong Kong Joint Laboratory for RNA Medicine, Medical Research Center, Sun Yat-sen Memorial Hospital, Sun Yat-sen University, Guangzhou, China

5. Department of Joint Surgery, the First Affiliated Hospital, Sun Yat-sen University, Guangzhou, China

6. Department of Rehabilitation, Zhujiang Hospital, Southern Medical University, Guangzhou, China

7. Key Laboratory of Stem Cells and Tissue Engineering, Zhongshan School of Medicine, Sun Yat-Sen University, Ministry of Education, Guangzhou, China

8. Department of Rehabilitation Medicine, the Sixth Affiliated Hospital, Sun Yat-sen University, Guangzhou, China

9. Department of Psychiatry, The Second Xiangya Hospital, Central South University, Changsha, Hunan, China

†These authors have contributed equally to this work

*Co-Corresponding authors: Xiaojing Ye, email: yexiaoj8@mail.sysu.edu.cn; Wei-Jye Lin, email: linwj26@mail.sysu.edu.cn; Yan Zhang, email: yan.zhang@csu.edu.cn

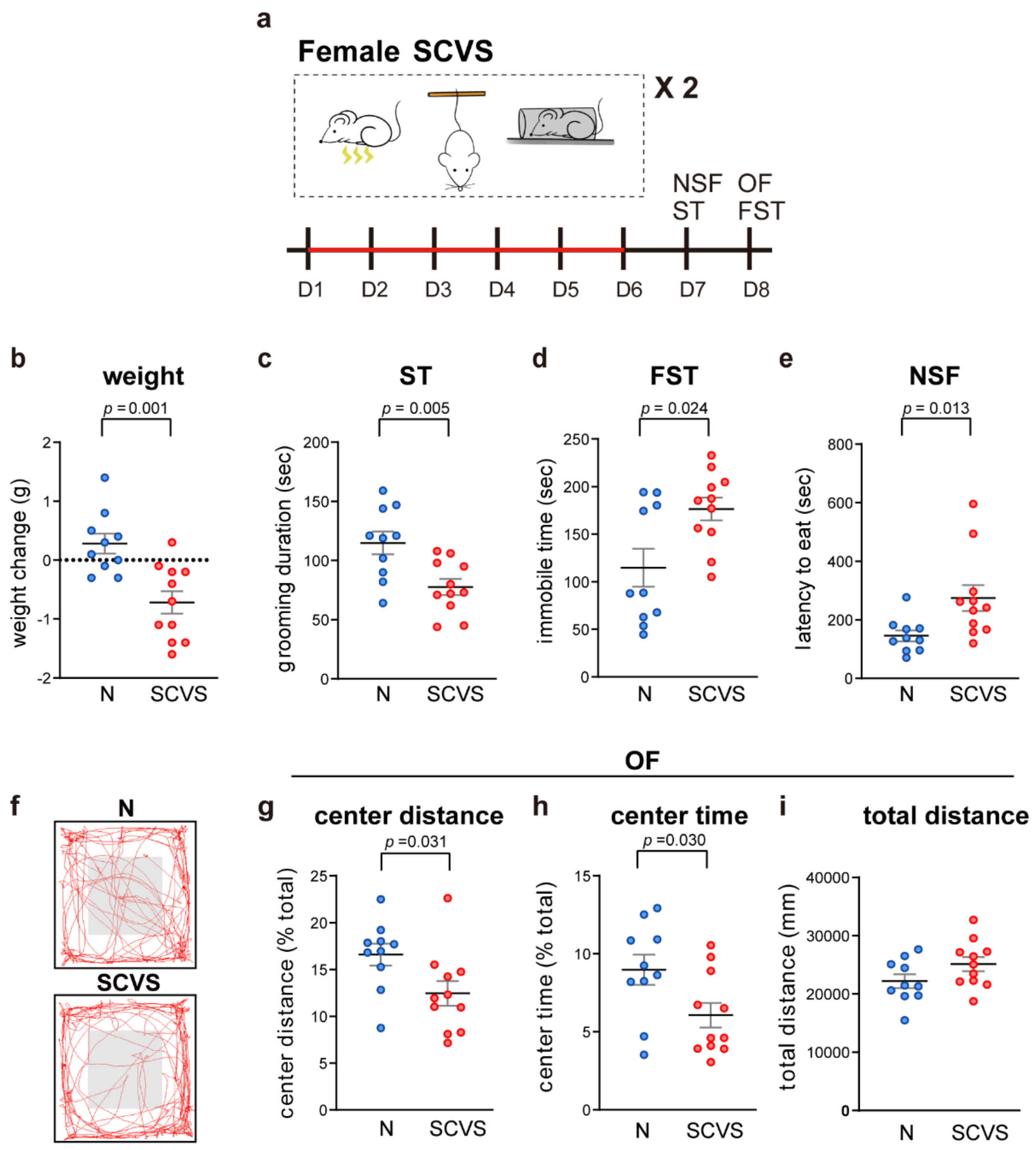

Supplementary Figure 1. Six days of sub-chronic variable stress (SCVS) induce depression- and anxiety-like behaviors in female mice. a The experimental timeline of the SCVS paradigm and behavioral tests. **b** Quantification of changes in the body weight by SCVS, compared with non-stressed naïve mice (N). **c** Quantification of grooming duration in the splash test (ST). **d** Quantification of the immobile time in the forced swim test (FST).

e Quantification of the latency to eat in the novelty-suppressed feeding test (NSF). **f** Representative traces of animal's paths in the open field (OF). The center zone is indicated by the grey box in the center. **g-i** Quantification of the travelled distance in the center zone as a percentage of the total travelled distance (**g**), the time spent in the center zone as a percentage of the total time (**h**), and total travelled distance (**i**) in the OF (**b-e, g-i:** n = 10-11 per group; results from two independent experiments). All data are presented as mean ± s.e.m. and analyzed by unpaired Student's *t* tests (**b, c, g-i**) or Mann Whitney tests (**d, e**). Source data are provided as a Source Data file.

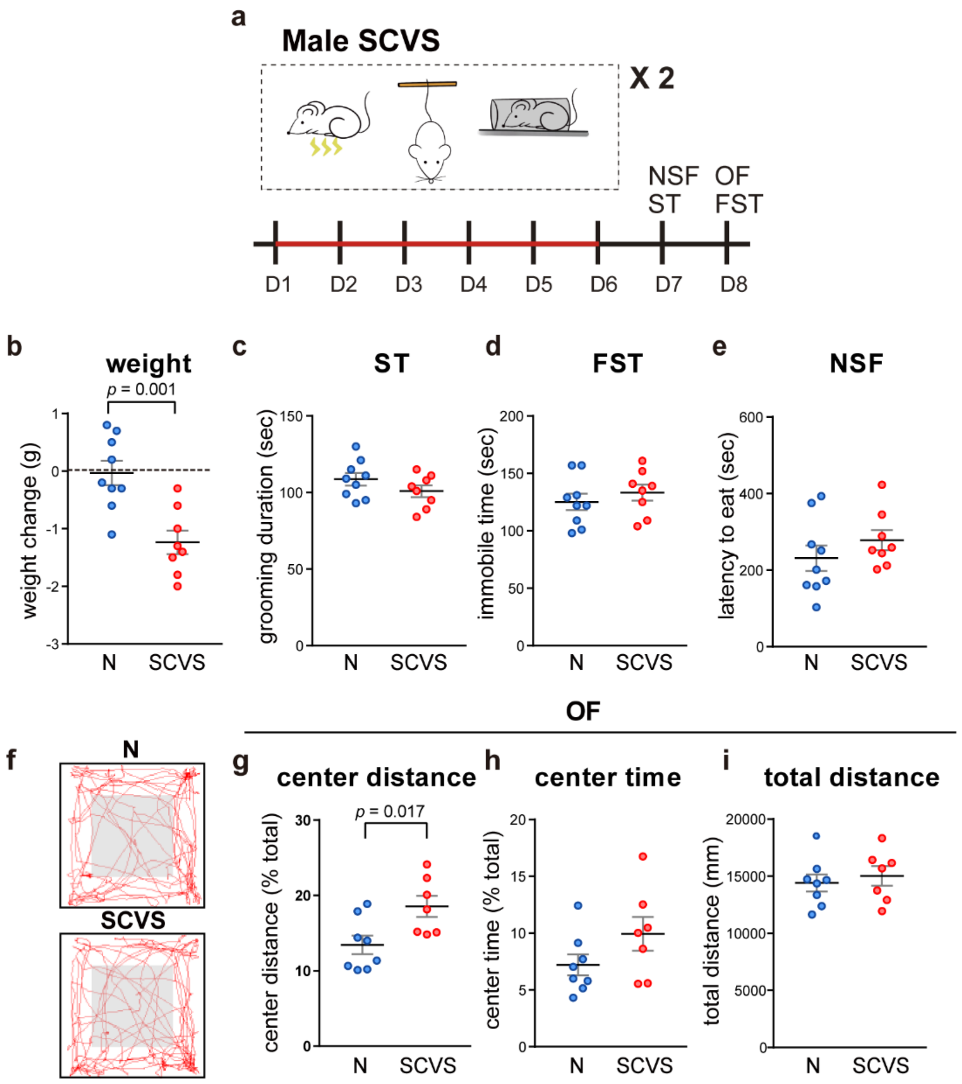

Supplementary Figure 2. Six days of sub-chronic variable stress (SCVS) do not induce depression- or anxiety-like behaviors in male mice. a The experimental timeline of the SCVS paradigm and behavioral tests. **b** Quantification of changes in the body weight by SCVS, compared with non-stressed naïve mice (N). **c** Quantification of grooming duration in the splash test (ST). **d** Quantification of the immobile time in the forced swim test (FST). **e** Quantification of the latency to eat in the novelty-suppressed feeding test (NSF). **f** Representative traces of animal's paths in the open field (OF). The center zone is indicated

by the grey box in the center. **g-i** Quantification of the travelled distance in the center zone as a percentage of the total travelled distance (**g**), the time spent in the center zone as a percentage of the total time (**h**), and total travelled distance (**i**) in the OF (**b-e:** n = 8-9 per group**; g-i:** n = 7-8 per group; results from two independent experiments). All data are presented as mean ± s.e.m. and analyzed by unpaired Student's *t* tests. Source data are provided as a Source Data file.

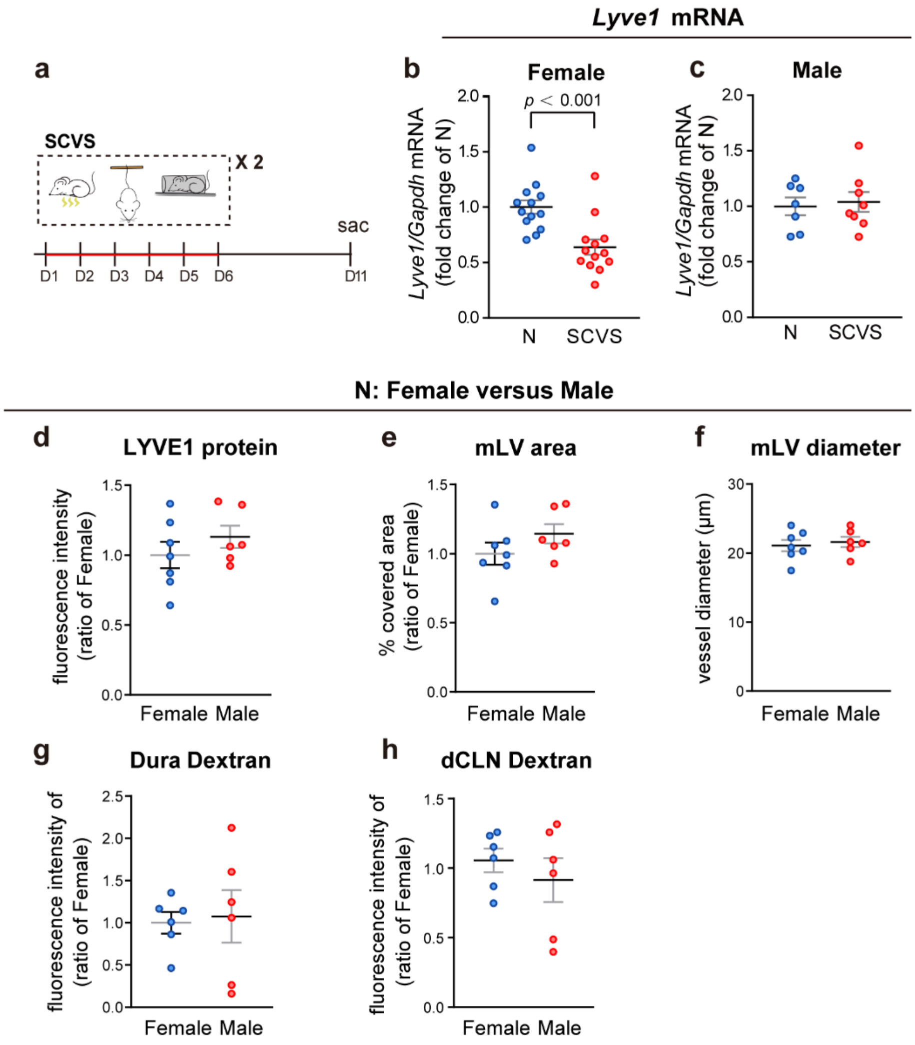

Supplementary Figure 3. Sex difference in changes of *Lyve1* mRNA expression in the dura mater by sub-chronic variable stress (SCVS), and comparison of meningeal lymphatics (mLV) in naïve (N) female and male mice. a The experimental timeline of the SCVS paradigm and tissue collection (sac). **b-c** Quantification of fold changes of the *Lyve1* mRNA levels in the dura mater of female (**b**: n = 13 per group; results from three independent experiments) and male (**c**: n = 7-8 per group; results from two independent experiments) mice, comparing SCVS *versus* N mice. **d-f** Quantification of the fluorescence intensity of the

LYVE1 staining (**d**), the area covered by mLV (**e**) and the diameter of LYVE1-labelled mLV (**f**) in the superior sagittal sinus (SSS) and the confluence of sinus and transverse sinus (COS+TS) areas of dura mater in the N female and male mice. **g** Quantification of the fluorescence intensity of the intracisternally-injected dextran tracer in the SSS and COS+TS areas of dura mater. **h** Quantification of the fluorescence intensity of dextran tracer in the dCLN (**d-h:** n = 6-7 per group; results from two independent experiments). All data are presented as mean ± s.e.m. and analyzed by unpaired Student's *t* tests. Source data are provided as a Source Data file.

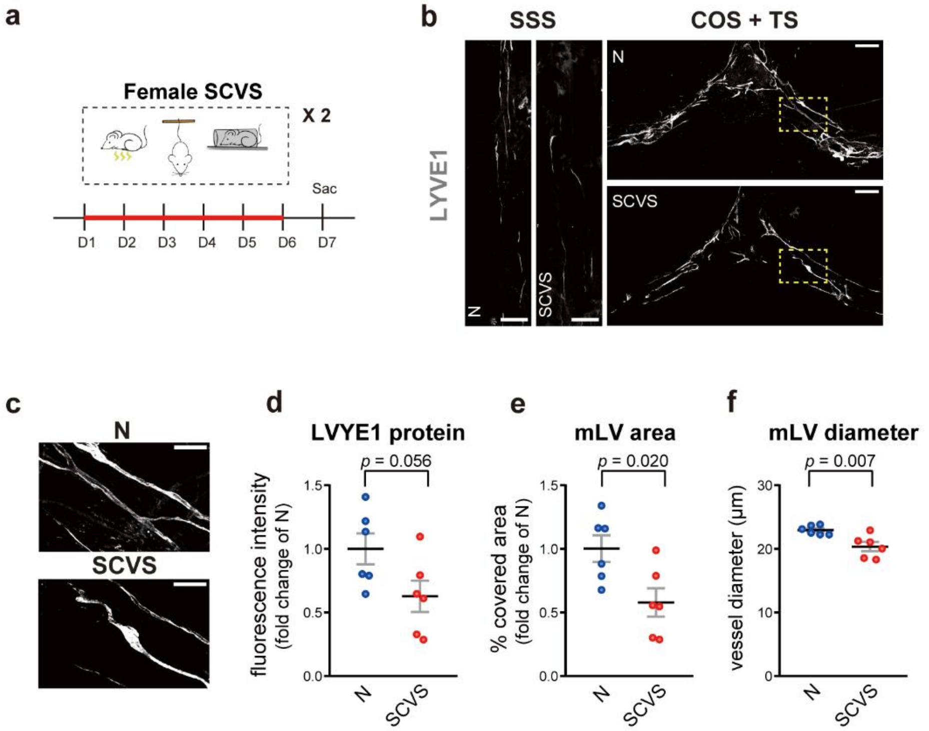

Supplementary Figure 4. Meningeal lymphatics in female mice are impaired at 24 h after sub-chronic variable stress (SCVS). a The experimental timeline of the SCVS paradigm and tissue collection (sac). **b** Representative images depicting the LYVE1 staining (grey) in the superior sagittal sinus (SSS) as well as the confluence of sinus and transverse sinus (COS+TS) areas of dura mater of female mice, comparing non-stressed naïve group (N) and the SCVS group. Scale bars: 500 µm. **c** Representative images depicting LYVE1-labelled meningeal lymphatic vessels (mLV) of female mice at higher magnification. Scale bars: 200 µm. **d-f** Quantification of the fluorescence intensity of the LYVE1 staining (**d**), the area covered by mLV (**e**) and the diameter of LYVE1-labelled mLV (**f**) in the SSS and COS+TS areas of dura mater in female mice (**d-f:** n = 6 per group; results from two independent experiments) mice. All data are presented as mean ± s.e.m. and analyzed by unpaired Student's *t* tests. Source data are provided as a Source Data file.

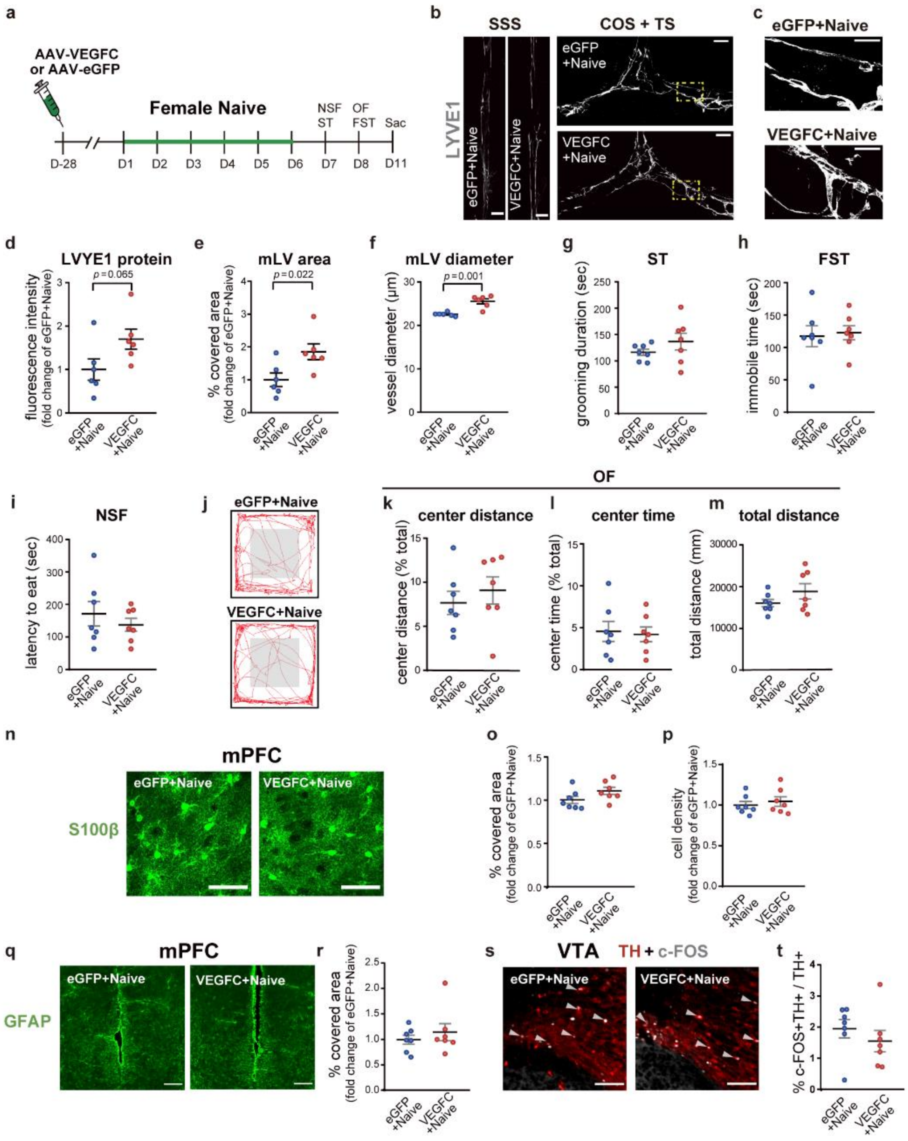

Supplementary Figure 5. Intracisternal delivery of AAV-VEGFC improves meningeal lymphatics without affecting depression-like behaviors as well as astrocytic protein expression in the mPFC and c-FOS expression in the VTA dopaminergic neurons of non-stressed female mice. a The experimental timeline of the intracisternal AAV infusion, behavioral tests and tissue collection (sac). **b** Representative images of the LYVE1 staining (grey) in the superior sagittal sinus (SSS) and the confluence of sinus and transverse sinus

(COS+TS) areas of dura mater, comparing female mice injected with AAV-VEGFC (VEGFC+Naïve) *versus* those injected with AAV-eGFP (eGFP+Naïve). Scale bars: 500 µm. **c** Representative images of LYVE1-labelled mLV at higher magnification. Scale bars: 200 µm. **d-f** Quantification of the fluorescence intensity of the LYVE1 staining (**d**), the area covered by mLV (**e**), and the diameter of LYVE1-labelled mLV (**f**). **g-i** Quantification of grooming duration in the splash test (ST, **g**), the immobile time in the forced swim test (FST, **h**) and the latency to eat in the novelty-suppressed feeding test (NSF, **i**). **j** Representative traces of animal's paths in the open field (OF). **k-m** Quantification of the travelled distance in the center zone as a percentage of the total travelled distance (**k**), the time spent in the center zone as a percentage of the total time (**l**), and total travelled distance (**m**) in the OF. **n, q** Representative images of the S100β (**n**: scale bars: 50 µm) and GFAP (**q**: scale bars: 200 µm) staining in the mPFC. **o, r** Quantification of the percentage of covered area by S100β (**o**) and GFAP (**r**) staining in mPFC. **p** Quantification of the density of S100β-labelled astrocytes in mPFC. **s** Representative images of the TH (red) and c-FOS (grey) staining in the VTA. White arrowheads denote cells dual-labelled by c-FOS and TH. Scale bars: 100 µm. **t** Quantification of the percentage of c-FOS$^+$ neurons in TH-labelled dopaminergic neurons in the VTA (**d-f:** n = 6 per group; **g-i, k-m, o-p, r, t:** n = 7 per group; results from two independent experiments). All data are presented as mean ± s.e.m. and analyzed by unpaired Student's *t* tests (**d-i, k-m, o-p**) or Mann Whitney tests (**r, t**). Source data are provided as a Source Data file.

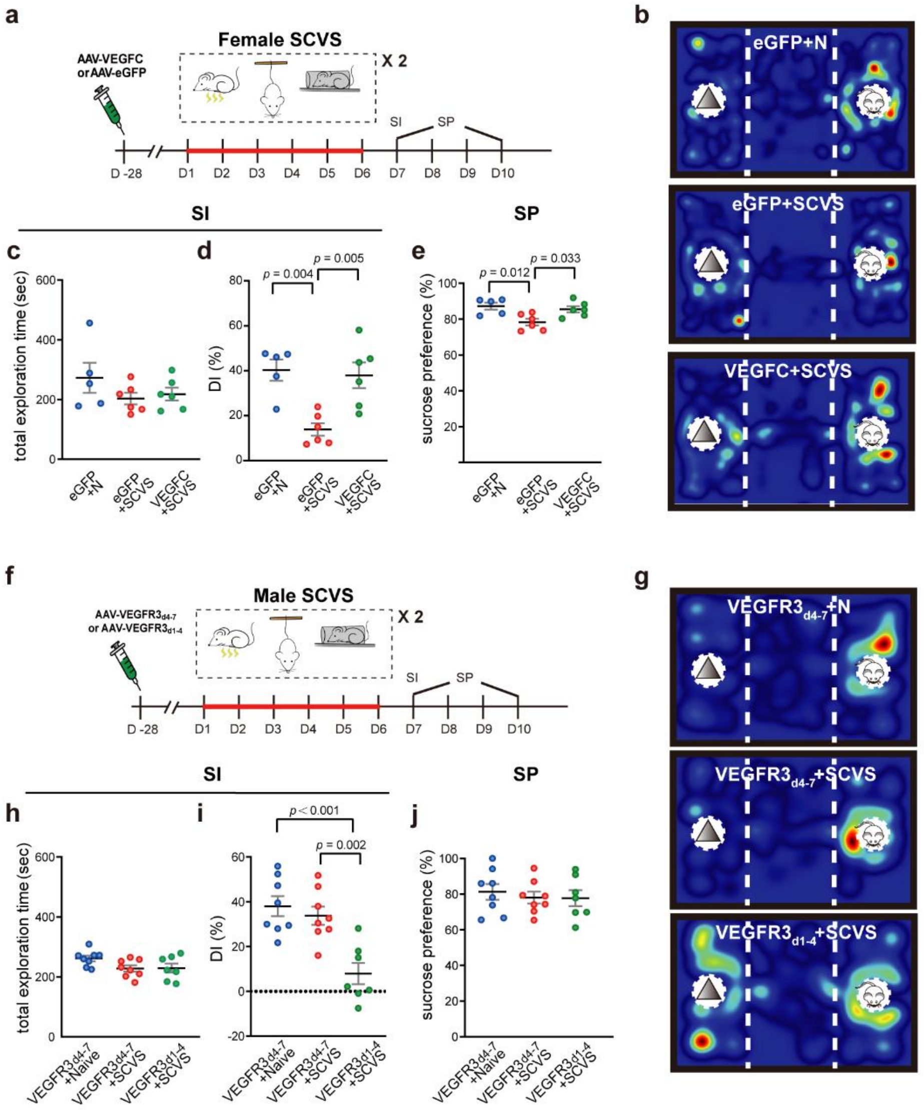

Supplementary Figure 6 Regulation of sub-chronic variable stress (SCVS)-induced impairment in social interaction and sucrose preference by manipulation of meningeal lymphatics. a,f The experimental timeline of the intracisternal AAV infusion, sub-chronic variable stress (SCVS) paradigm, behavioral tests for social interaction (SI) and sucrose preference (SP) of female (**a**) and male (**f**) mice. **b, g** Representative heatmaps of female (**b**) and male (**g**) mice's trace in SI. **c-d** Quantification of the total exploration time and

differential index (DI) in the SI, comparing naïve female mice intracisternally injected with AAV-eGFP (eGFP+N), female mice injected with AAV-eGFP and experienced SCVS (eGFP+SCVS) and female mice injected with AAV-VEGFC and experienced SCVS (VEGFC+SCVS). **e** Quantification of sucrose preference between the three groups of female mice (**c-e:** n = 5-6 per group; results from two independent experiments). **h-i** Quantification of the total exploration time and DI in the SI, comparing naïve male mice intracisternally injected with AAV-$VEGFR3_{d4-7}$ ($VEGFR3_{d4-7}$+N), male mice injected with AAV- $VEGFR3_{d4-7}$ and experienced SCVS ($VEGFR3_{d4-7}$+SCVS) and male mice injected with AAV-$VEGFR3_{d1-4}$ and experienced SCVS ($VEGFR3_{d1-4}$+SCVS). **j** Quantification of sucrose preference between the three groups of male mice (**h-j:** n = 7-8 per group; results from two independent experiments). All data are presented as mean ± s.e.m. and analyzed by one-way ANOVA followed by Tukey's *post hoc* tests. Source data are provided as a Source Data file.

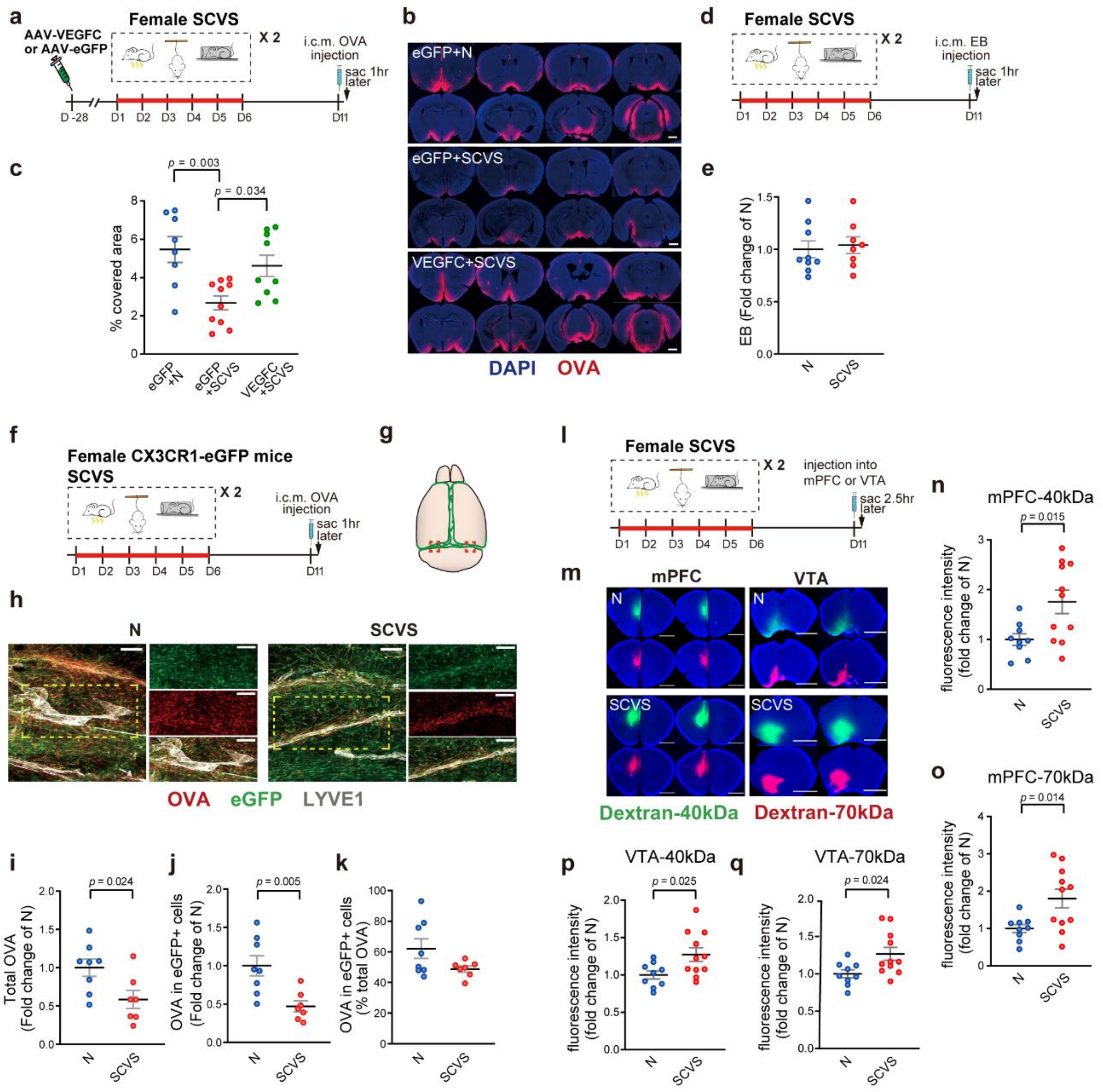

Supplementary Figure 7 Distribution of intracisternally- or intracerebrally-injected tracers in the brain of the female mice after sub-chronic variable stress (SCVS). a, d, f The experimental timeline of the intracisternal AAV infusion, sub-chronic variable stress (SCVS) paradigm, the intracisternal (i.c.m.) ovalbumin-Alexa Fluor 647 tracer (OVA) or Evans Blue (EB) injection and tissue collection (sac). **b, c** Representative images (**b**) and quantification (**c**) of intracisternally-injected OVA (red) distribution in the brain sections, comparing naïve mice injected with AAV-eGFP (eGFP+N), mice injected with AAV-eGFP

and experienced SCVS (eGFP+SCVS) and mice injected with AAV-VEGFC and experienced SCVS (eGFP+SCVS). Nuclei were counterstained with DAPI (blue). Scale bars: 1000 µm. (n = 8-10 per group; results from three independent experiments). **e** Quantification of the distribution of intracisternally-injected EB tracer in the brain homogenate, comparing naïve (N) female mice and those experienced SCVS (n = 8-9 per group; results from two independent experiments). **g** The schematic diagram of dura mater, with dotted red line bordering the transverse sinus (TS) areas chosen for image analyses. **h** Representative images of immunofluorescence staining of LYVE1 (grey), OVA (red) and eGFP-labelled macrophages (green) in the TS area of dura mater. Scale bars: 100 µm. **i-k** Quantification of the percentage of covered area of total OVA (**i**), OVA in eGFP-labelled macrophages (**j**) and OVA in eGFP-labelled macrophages as a percentage of total OVA (**k**) in the TS area of dura mater (**i-k:** n = 7-8 per group; results from two independent experiments). **l** The experimental timeline of SCVS paradigm, the injection of 40kDa and 70kDa dextran tracer into the mPFC and VTA, and tissue collection (sac). **m** Representative images of tracer distribution in the mPFC and VTA, comparing N and SCVS groups. Scale bars: 500 µm. **n-q** Quantification of the 40kDa (**n, p**) or 70kDa (**o, q**) dextran tracer left in the mPFC (**n, o**) or VTA (**p, q**) at 2.5 h post-injection (n = 9-11 per group; results from three independent experiments). All data are presented as mean ± s.e.m. and analyzed by one-way ANOVA followed by Tukey's *post hoc* tests or unpaired Student's *t* tests. Source data are provided as a Source Data file.

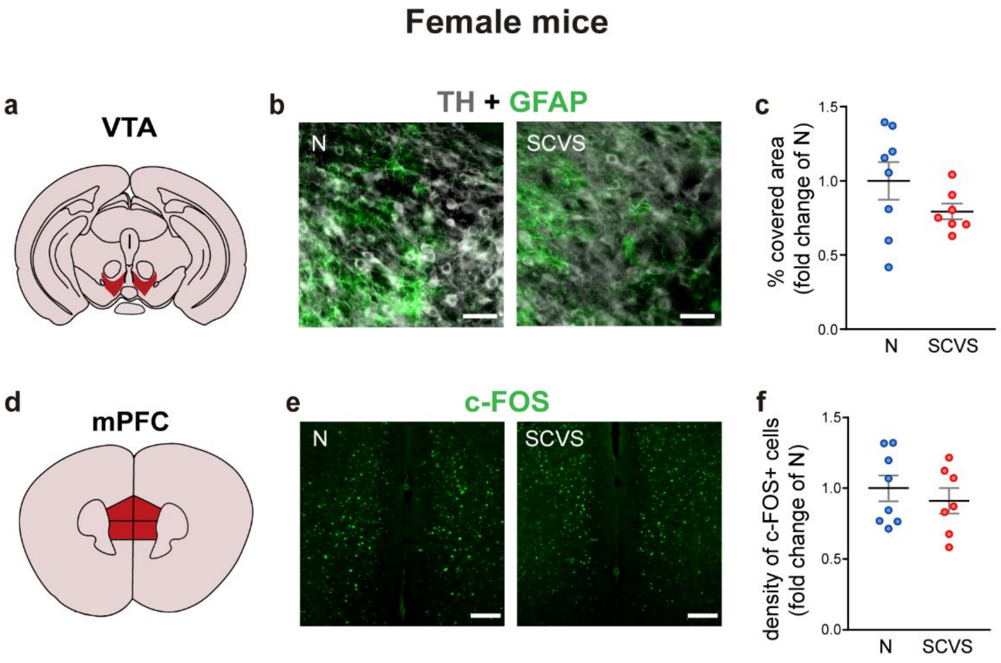

Supplementary Figure 8 Sub-chronic variable stress (SCVS) does not significantly alter GFAP expression in the VTA or c-FOS expression in the mPFC of female mice. a The coronal atlas with the VTA highlighted in red. **b-c** Representative images (**b**: scale bars = 50 µm) and quantification (**c**) of GFAP (green) immunofluorescence staining in the VTA, comparing female mice experienced SCVS with non-stressed naïve mice (N). Dopaminergic neurons were co-stained with TH (grey). **d** The coronal atlas with the mPFC highlighted in red. **e-f** Representative images (**e**: scale bars: 200 µm.) and quantification (**f**) of c-FOS immunofluorescence staining in the mPFC, comparing SCVS female mice with non-stressed naïve mice (N). (**c, f:** n = 7-8 mice per group; results from two independent experiments). All data are presented as mean ± s.e.m. and analyzed by unpaired Student's *t* tests. Source data are provided as a Source Data file.

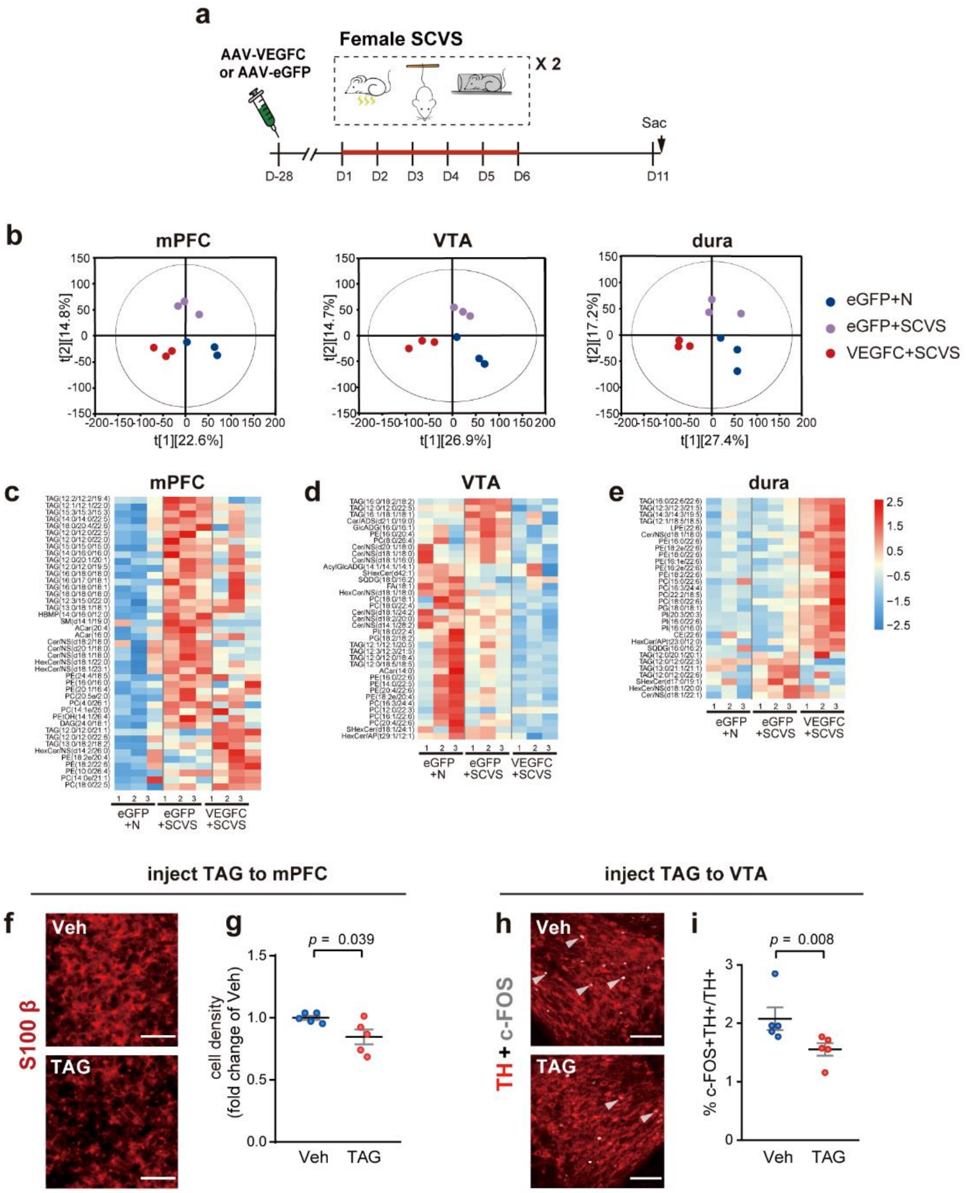

Supplementary Figure 9 Sub-chronic variable stress (SCVS) and intracisternal delivery of AAV-VEGFC alter lipidomic profiles of the mPFC, the VTA and the dura mater of female mice. a The experimental timeline of the AAV infusion, SCVS paradigm, and tissue collection (sac). **b** The partial least squares discriminant analysis (PLS-DA) score plots of the lipidomic profiles in the mPFC, the VTA and the dura mater, comparing naïve mice

injected with AAV-eGFP (eGFP+N), mice injected with AAV-eGFP and experienced SCVS (eGFP+SCVS), and mice injected with AAV-VEGFC and experienced SCVS (VEGFC+SCVS). **c-e** Heatmaps showing relative expression levels of significantly changed lipid species (VIP > 1 and $p < 0.05$) in the three groups of samples. Color scale bar values represent standardized rlog-transformed values across samples (3 biological replicates per group, each pooled from samples collected from 4 animals). **f** Representative images of the S100β staining in the mPFC. Scale bars: 100 µm. **g** Quantification of the density of S100β-labelled astrocytes in the mPFC. **h** Representative images of the TH (red) and c-FOS (grey) staining in the VTA. White arrowheads denote cells dual-labelled by c-FOS and TH. Scale bars: 100 µm. **i** Quantification of the percentage of c-FOS^{+} neurons in TH-labelled dopaminergic neurons in the VTA (**g, i:** n = 5 per group; results from two independent experiments). All data are presented as mean ± s.e.m. and analyzed by unpaired Student's *t* tests (**g**) or Mann Whitney tests (**i**). Source data are provided as a Source Data file.

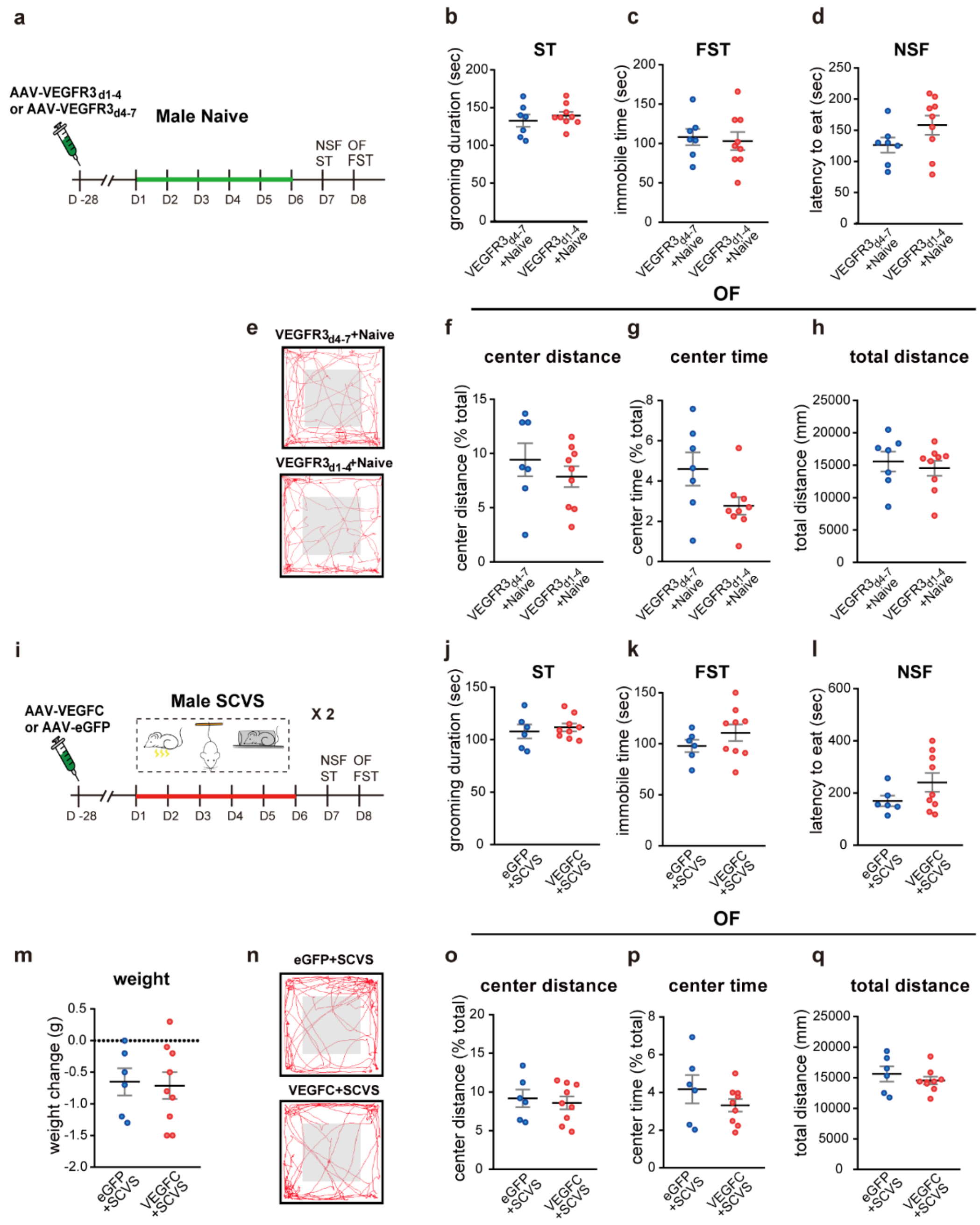

Supplementary Figure 10 Intracisternal delivery of AAV-VEGFR3d1-4 in naïve male mice or AAV-VEGFC in male mice experienced sub-chronic variable stress (SCVS) does not alter depression- and anxiety-like behaviors. a The experimental timeline of intracisternal AAV infusion and behavioral tests. **b-d** Quantification of grooming duration in

the splash test (ST, **b**), the immobile time in the forced swim test (FST, **c**) and the latency to eat in the novelty-suppressed feeding test (NSF, **d**), comparing naïve male mice intracisterally injected with AAV-VEGFR3$_{d4-7}$ as control (VEGFR3$_{d4-7}$+Naïve) or AAV-VEGFR3$_{d1-4}$ (VEGFR3$_{d1-4}$+Naïve). **e** Representative traces of animal's paths in the open field (OF). **f-h** Quantification of the travelled distance in the center zone as a percentage of the total travelled distance (**f**), the time spent in the center zone as a percentage of the total time (**g**), and total travelled distance (**h**) in the OF (**b-d, f-h:** n = 7-9 per group; results from two independent experiments). **i** The experimental timeline of intracisternal AAV infusion, SCVS and behavioral tests. **j-l** Quantification of grooming duration in the ST (**j**), the immobile time in the FST (**k**) and the latency to eat in the NSF (**l**), comparing male mice intracisterally injected with AAV-eGFP as control (eGFP+SCVS) or AAV-VEGFC (VEGFC+SCVS). Both groups of male mice experienced SCVS. **m** Quantification of changes in the body weight by SCVS. **n** Representative traces of animal's paths in the OF. **o-q** Quantification of the travelled distance in the center zone as a percentage of the total travelled distance (**o**), the time spent in the center zone as a percentage of the total time (**p**), and total travelled distance (**q**) in the OF (**j-m, o-q:** n = 6-9 per group; results from two independent experiments). All data are presented as mean ± s.e.m. and analyzed by unpaired Student's *t* tests. Source data are provided as a Source Data file.

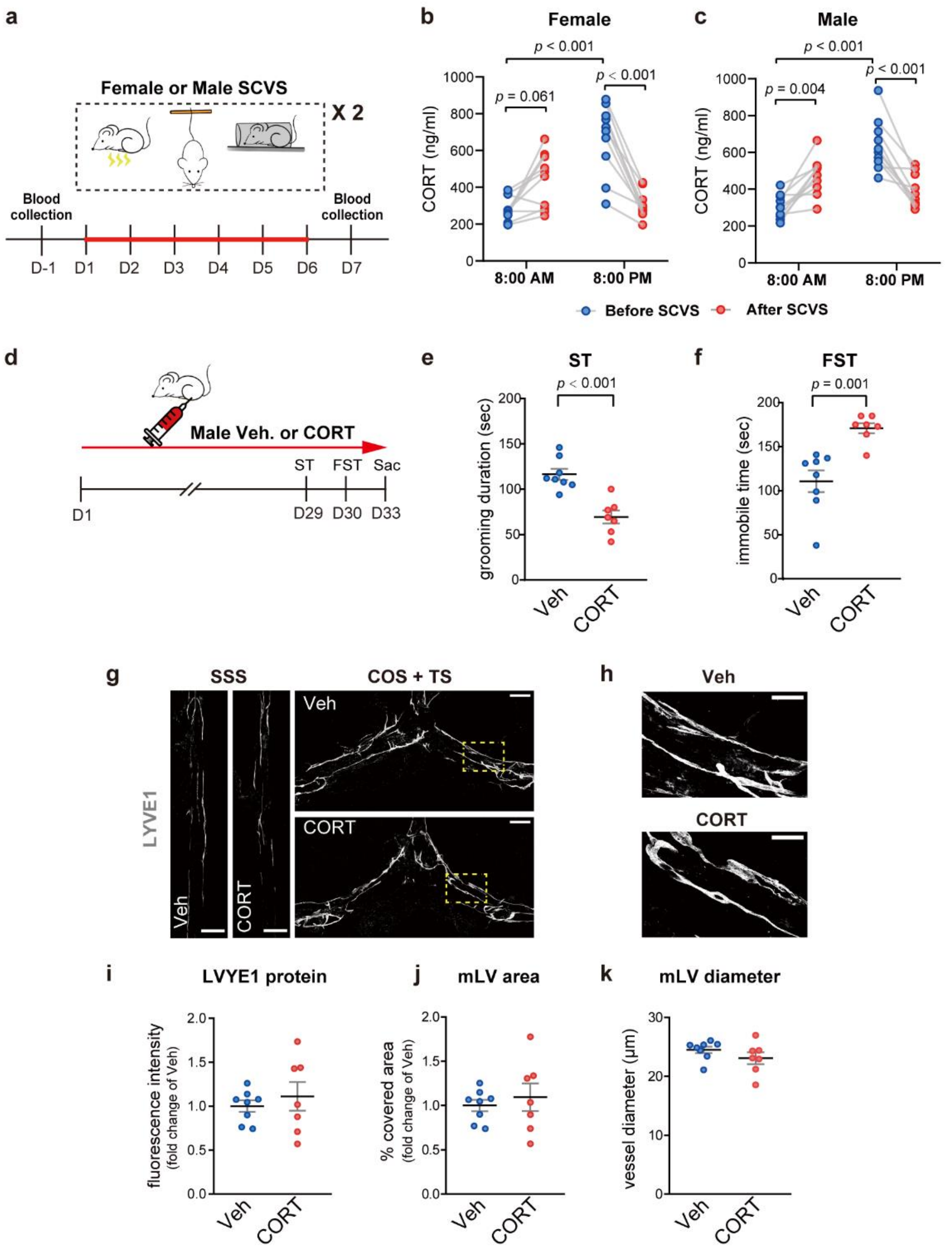

Supplementary Figure 11 Corticosterone (CORT) is not involved in sex-different impairment of meningeal lymphatics by sub-chronic variable stress (SCVS). a The experimental timeline of the SCVS paradigm and blood collection. **b**-**c** Quantification of serum CORT concentration at 8:00 AM and 8:00 PM before and after SCVS in female (**b**)

and male (**c**) mice (n = 10 per group; results from two independent experiments). **d** The experimental timeline of intraperitoneal injection of vehicle (Veh) or CORT into male mice, behavioral tests and tissue collection (sac). **e** Quantification of grooming duration in the splash test (ST). **f** Quantification of the immobile time in the forced swim test (FST). **g** Representative images of the LYVE1 staining (grey) in the SSS and COS+TS areas of dura mater, comparing male mice injected with vehicle and CORT. Scale bars: 500 µm. **h** Representative images depicting LYVE1-labelled mLV at higher magnification. Scale bars: 200 µm. **i-k** Quantification of the fluorescence intensity of the LYVE1 staining (**i**), the area covered by mLV (**j**) and the diameter of LYVE1-labelled mLV (**k**). (**e-f, i-k:** n = 7-8 per group; results from two independent experiments). All data are presented as mean ± s.e.m. and analyzed by two-way ANOVA followed by Tukey's *post hoc* tests (**b-c**) or unpaired Student's *t* tests (**e-f, i-k**). Source data are provided as a Source Data file.
