# Supplementary material for: A functional role of meningeal lymphatics in sex difference of stress susceptibility in mice
Source: Nat Commun. 2022 Aug 16;13:4825. doi: 10.1038/s41467-022-32556-x (PMC9381547; doi:10.1038/s41467-022-32556-x)
Supplement: Supplementary file 3 — Reporting Summary [file 41467_2022_32556_MOESM3_ESM.pdf]

nature portfolio

Corresponding author(s): Xiaojing Ye, PhD; Wei-Jye Lin, PhD; Yan Zhang, MD

Last updated by author(s): Jul 10, 2022

Reporting Summary

Nature Portfolio wishes to improve the reproducibility of the work that we publish. This form provides structure for consistency and transparency in reporting. For further information on Nature Portfolio policies, see our Editorial Policies and the Editorial Policy Checklist.

Statistics

For all statistical analyses, confirm that the following items are present in the figure legend, table legend, main text, or Methods section.

n/a	Confirmed	
☐	☒	The exact sample size (*n*) for each experimental group/condition, given as a discrete number and unit of measurement
☐	☒	A statement on whether measurements were taken from distinct samples or whether the same sample was measured repeatedly
☐	☒	The statistical test(s) used AND whether they are one- or two-sided<br>*Only common tests should be described solely by name; describe more complex techniques in the Methods section.*
☐	☒	A description of all covariates tested
☐	☒	A description of any assumptions or corrections, such as tests of normality and adjustment for multiple comparisons
☐	☒	A full description of the statistical parameters including central tendency (e.g. means) or other basic estimates (e.g. regression coefficient) AND variation (e.g. standard deviation) or associated estimates of uncertainty (e.g. confidence intervals)
☐	☒	For null hypothesis testing, the test statistic (e.g. F, t, r) with confidence intervals, effect sizes, degrees of freedom and P value noted<br>*Give P values as exact values whenever suitable.*
☒	☐	For Bayesian analysis, information on the choice of priors and Markov chain Monte Carlo settings
☒	☐	For hierarchical and complex designs, identification of the appropriate level for tests and full reporting of outcomes
☒	☐	Estimates of effect sizes (e.g. Cohen's d, Pearson's r), indicating how they were calculated

Our web collection on statistics for biologists contains articles on many of the points above.

Software and code

Policy information about availability of computer code

Data collection	When possible, behavioral tests were recorded and analyzed using EthoVision XT software (version 15.0.1416, Noldus, Netherlands) and DigBehv software (version 4.1.7.171129, Ji-Liang, Shanghai, China).

Data analysis	Statistical analysis and graph generation: GraphPad Prism (version 7.0).<br>Immunofluorescent image: ImageJ (version 1.52n).<br>FACS and data analysis: MoFlo Astrios EQs (version Summit 6.2), FlowJo software (version 10).<br>RNA-seq data analyses: The processed sequences were aligned to the mm9 mouse reference genome (UCSC) using Hisat2 (version 2.0.1). Statistical analysis was performed using DEseq2 (version 1.32.0). The heatmap (pheatmap, version 1.0.12) and volcano plot of the DEGs were generated with pheatmap and plot packages in R (version 4.1.0). The gene-set enrichment analysis (GSEA) was performed to identify KEGG pathways (c2.cp.kegg.v7.4.symbols.gmt) and gene ontology (GO, c5.go.bp.v7.4.symbols.gmt, c5.go.mf.v7.4.symbols.gmt and c5.go.cc.v7.4.symbols.gmt) that were differentially enriched between groups. The top 10 gene sets enriched in each group were plotted with ggplot2 (version 3.3.5) in R (version 4.1.0).<br>Lipidomic analyses: the raw data files were converted to files in mzXML format using the 'msconvert' program from ProteoWizard. The CentWave algorithm in XCMS (version 3.2) was used for peak detection, extraction, alignment, and integration. Lipid identification was achieved through a spectral match using LipidBlast library, which was developed using R and based on XCMS (version 3.2). The Partial least squares discrimination analysis (PLS-DA) was used to determine lipid differences between the three groups. For paired comparison, the score plots of Orthogonal Partial Least Squares-Discriminant Analysis (OPLS-DA) model was generated on the positive- and negative-mode MS data obtained from the XCMS program, which had been centered and scaled to unit variance scaling. Both PLS-DA and OPLS-DA were performed using the SIMCA (version 16.0.2, Sartorius Stedim Data Analytics AB, Umea, Sweden). Differentially changed lipid were visualized as heatmaps using the pheatmap (version 1.0.12) package in R (version 4.1.0).

For manuscripts utilizing custom algorithms or software that are central to the research but not yet described in published literature, software must be made available to editors and reviewers. We strongly encourage code deposition in a community repository (e.g. GitHub). See the Nature Portfolio guidelines for submitting code & software for further information.

Data

Policy information about availability of data

All manuscripts must include a data availability statement. This statement should provide the following information, where applicable:

- Accession codes, unique identifiers, or web links for publicly available datasets
- A description of any restrictions on data availability
- For clinical datasets or third party data, please ensure that the statement adheres to our policy

The RNA sequencing data generated for this study can be found in the GEO repository under accession number GSE201368 (https://www.ncbi.nlm.nih.gov/geo/query/acc.cgi?acc=GSE201368). Source data are provided as a Source Data file. Further information of this study is available upon reasonable request from the corresponding authors Xiaojing Ye (yexiaoj8@mail.sysu.edu.cn), Wei-Jye Lin (linwj26@mail.sysu.edu.cn) and Yan Zhang (yan.zhang@csu.edu.cn)

Human research participants

Policy information about studies involving human research participants and Sex and Gender in Research.

Reporting on sex and gender	No human research participants were included in this study.
Population characteristics	No human research participants were included in this study.
Recruitment	No human research participants were included in this study.
Ethics oversight	No human research participants were included in this study.

Note that full information on the approval of the study protocol must also be provided in the manuscript.

Field-specific reporting

Please select the one below that is the best fit for your research. If you are not sure, read the appropriate sections before making your selection.

☒ Life sciences ☐ Behavioural & social sciences ☐ Ecological, evolutionary & environmental sciences

For a reference copy of the document with all sections, see nature.com/documents/nr-reporting-summary-flat.pdf

Life sciences study design

All studies must disclose on these points even when the disclosure is negative.

Sample size	Sample size for subchronic variable stress (SCVS) mouse cohorts were calculated based on previous studies of SCVS and depression-like behaviors (Zhang S, et al., 2018; Jiang C, et al., 2019). Sample size for intracisternal injection experiments was determined based on the previous studies of meningeal lymphatics (Da Mesquita S, et al.,2018; Xavier ALR, et al., 2018). For RNA-seq and metabolomic analysis, no statistical method was used and sample size was determined based on similar experiments conducted previously (Rustenhoven J, et al.,2021).
Data exclusions	Outliers in the behavioral datasets which passed Shapiro-Wilk normality test were identified as being greater than 2 standard deviations from the mean, and were excluded from statistical analysis as described previously (Dion-Albert L, et al., 2022).

Replication	All experiments (behavioral tests, immunofluorescence and tracer quantification, RNA-seq analysis, and metabolomics analysis) were performed with at least two cohorts of mice to ensure reproducibility of the results. When automatic tracking was not available, data were analyzed by the observers blinded to the experimental conditions to avoid bias.
Randomization	Mice were randomized upon arrival and were randomly assigned to different groups immediately before the start of the experiments.
Blinding	The open field tests were performed with automated tracking systems. Scoring for other behavioral experiments was done by experimenters blinded to experimental conditions (for splash test, sucrose preference test, novelty suppressed feeding, social interaction test and forced swim test). For immunofluorescence and tracer quantification, sample IDs were renamed to render the experimenter 'blind', and true subject IDs were revealed once data analysis was finished.

Reporting for specific materials, systems and methods

We require information from authors about some types of materials, experimental systems and methods used in many studies. Here, indicate whether each material, system or method listed is relevant to your study. If you are not sure if a list item applies to your research, read the appropriate section before selecting a response.

Materials & experimental systems

n/a	Involved in the study
☐	☒ Antibodies
☒	☐ Eukaryotic cell lines
☒	☐ Palaeontology and archaeology
☐	☒ Animals and other organisms
☒	☐ Clinical data
☒	☐ Dual use research of concern

Methods

n/a	Involved in the study
☒	☐ ChIP-seq
☐	☒ Flow cytometry
☒	☐ MRI-based neuroimaging

Antibodies

Antibodies used

The following antibodies were used in the study.
anti-c-FOS (IF, 1:500, cat# 2250, lot# 12, clone# 9F6, Cell Signaling Technologies, Danvers, MA, USA);
anti-GFAP (IF, 1:5000, cat# ab4674, lot# GR3349945-1, Abcam, Biomedical Campus,Cambridge, UK);
anti-TH (IF, 1:8000, cat# MAB7566, lot# CGUO0120071, clone# 779427, R&D Systems, Emeryville, CA, USA);
anti-LYVE1 (IF, 1:500, cat# ab14917, lot# GR3392340-6, Abcam, Biomedical Campus,Cambridge, UK);
anti-S100β (IF, 1:500, cat# ab52642, lot# GR3215095-15, clone# EP1576Y, Abcam, Biomedical Campus,Cambridge, UK);
anti-CD45–BB515 (FACS, 1:200, cat# 564590, lot# 0216978, clone# 30-F11, BD Bioscience, San Jose, California, USA);
anti-CD31-Alexa Fluor 647 (FACS, 1:200, cat# 563608, lot# 9199302, clone# 390, BD Bioscience, San Jose, California, USA);
anti-Podoplanin-PE (FACS, 1:200, cat# 12-5381-82, lot# 2120142, clone# eBio8.1.1, eBioscience, San Diego, California, USA);
anti-CCL6 (Neutralization, 1 μg/μL, cat# MAB487, lot# UPR0221021, clone# 262016, R&D system, USA);
anti-IgG (Neutralization, 1 μg/μL, cat# MAB0061, lot# HBI1021101, clone# 141945, R&D system, USA).

Validation

Antibodies used in this studies were all reported and validated by the manufacturer and in the literature.
-c-FOS (Rabbit mAb, IF validated, cat# 2250, Cell Signaling Technologies, Danvers, MA, USA) https://www.cellsignal.cn/products/primary-antibodies/c-fos-9f6-rabbit-mab/2250?_=1657204754059&Ntt=2250&tahead=true
-GFAP (Chicken pAb, IHC validated, cat# ab4674, Abcam, Biomedical Campus,Cambridge, UK) https://www.abcam.cn/gfap-antibody-ab4674.html
TH (Mouse mAB, IHC validated, cat# MAB7566, R&D Systems, Emeryville, CA, USA) https://www.rndsystems.com/cn/products/human-mouse-tyrosine-hydroxylase-antibody-779427_mab7566
-LYVE1 (Rabbit pAb, IHC validated, cat# ab14917, Abcam, Biomedical Campus,Cambridge, UK) https://www.abcam.cn/lyve1-antibody-bsa-and-azide-free-ab14917.html
-S100β (Rabbit mAb, IF validated, cat# ab52642, Abcam, Biomedical Campus,Cambridge, UK) https://www.abcam.cn/s100-beta-antibody-ep1576y-astrocyte-marker-ab52642.html
-CD45–BB515 (Rat mAB, FACS validated, cat# 564590, BD Bioscience, San Jose, California, USA) https://www.bdbiosciences.com/zh-cn/search-results?searchKey=564590
-CD31-Alexa Fluor 647 (Rat mAB, FACS validated, cat# 563608, BD Bioscience, San Jose, California, USA) https://www.bdbiosciences.com/zh-cn/search-results?searchKey=563608
-Podoplanin-PE (Hamster mAB, FACS validated, cat# 12-5381-82, eBioscience, San Diego, California, USA) https://www.thermofisher.cn/cn/zh/antibody/product/Podoplanin-Antibody-clone-eBio8-1-1-8-1-1-Monoclonal/12-5381-82
-CCL6 (Mouse mAB, Neutralization validated, cat# MAB487, R&D system, USA) https://www.rndsystems.com/cn/products/mouse-ccl6-c10-antibody-262016_mab487
-IgG (Rat mAB, cat# MAB0061, R&D system, USA) https://www.rndsystems.com/cn/products/rat-igg-2b-isotype-control_mab0061

Animals and other research organisms

Policy information about studies involving animals; ARRIVE guidelines recommended for reporting animal research, and Sex and Gender in Research

Laboratory animals	Three months old female and male C57BL/6J mice, and female CX3CR1-GFP transgenic mice (stock #005582; the Jackson Laboratory), were used for the experiments. The sex of animals used in each experiment was specified in the figure legends. The animals were housed in groups of 4-5 in an environmentally controlled (temperature: 23 ± 2°C, humidity: 50–60%) animal facility on a 12 h/12 h dark/light cycle, with access to food and water ad libitum. All animal studies were reviewed and approved by the Institutional Animal Care and Use Committee of Sun Yat-sen University (Approval number: SYSU-IACUC-2019-000010).
Wild animals	No wild animals were used in the study.
Reporting on sex	The sex of the animals used for each experiments were reported in the Results section and Figure legends.
Field-collected samples	No field collected samples were used in the study.
Ethics oversight	All animal studies were reviewed and approved by the Institutional Animal Care and Use Committee of Sun Yat-sen University.

Note that full information on the approval of the study protocol must also be provided in the manuscript.

Flow Cytometry

Plots

Confirm that:

- [x] The axis labels state the marker and fluorochrome used (e.g. CD4-FITC).
- [x] The axis scales are clearly visible. Include numbers along axes only for bottom left plot of group (a 'group' is an analysis of identical markers).
- [x] All plots are contour plots with outliers or pseudocolor plots.
- [x] A numerical value for number of cells or percentage (with statistics) is provided.

Methodology

Sample preparation	Mice transcardially perfused with ice-cold PBS for 1 min under anesthesia. Individual meninx was quickly dissected from the skullcap in DMEM with 2% FBS (Thermo Scientific, Waltham, MA, US), and digested in preheated DMEM with 2% FBS, 1 mg/mL collagenase VIII, 0.5 mg/mL DNase I (Sigma-Aldrich, St. Louis, MO), 45 µM actinomycin D (Selleck, Houston, TX, US) for 12 min at 37°C. At the end of the digestion, 1 mL DMEM with 10% FBS were added to the solution to terminate digestion. Individual samples consisted of cell suspensions pooled from 3-4 meninges were filtered through a 70 µm nylon-mesh filter and washed with ice-cold fluorescence-activated cell sorting (FACS) buffer (PBS free of calcium and magnesium, 1 mM EDTA and 1% BSA, pH 7.4). The cells were pelleted by centrifugation at 400 g, 4°C for 5min, resuspended in 400 µL FACS buffer with anti-CD45–BB515 (BD Biosciences, clone 30-F11, 1:200), anti-CD31-Alexa Fluor 647 (BD Biosciences, clone 390, 1:200), anti-Podoplanin-PE (eBioscience, clone 8.1.1, 1:200) and DAPI (Sigma, 0.2 µg/mL), and incubated for 15 min at 4°C. Cells were then washed and resuspended in ice-cold FACS buffer.
Instrument	MoFlo Astrios EQs (Beckman Coulter, Indianapolis, IN, USA)
Software	FlowJo software (Tree Star, Ashland, OR, USA).
Cell population abundance	CD45- cells was 88.5%.<br>CD45+ cells was 10.1%.<br>Meningeal lymphatic endothelial cells was 0.42%.<br>Blood endothelial cells was 5.44%.<br>Other stromal cells was 84.3%.
Gating strategy	Meningeal lymphatic endothelial cells gated as CD45-PDPN+CD31+.<br>Blood endothelial cells gated as CD45-PDPN-CD31+.<br>CD45+ leukocytes gated as CD45+.<br>Other stromal cells gated as CD45-CD31-.

- [x] Tick this box to confirm that a figure exemplifying the gating strategy is provided in the Supplementary Information.
